# Data on how abundance of resource inflows and punishment types affect resource extraction behavior

**DOI:** 10.1016/j.dib.2023.109215

**Published:** 2023-05-09

**Authors:** Anna Lou Abatayo, John Lynham

**Affiliations:** a Environmental Economics and Natural Resources Group, Wageningen University and Research, Hollandseweg 1, 6706KN Wageningen, the Netherlands; b Department of Economics, University of Hawai**ʻ**i at Mãnoa, 2424 Maile Way, Honolulu, HI 96822, USA; c UHERO, University of Hawai**ʻ**i at Mānoa, 2424 Maile Way, Honolulu, HI, 96822, USA

**Keywords:** Resource curse, Common pool resources, Social ostracism, Financial Punishment

## Abstract

The data is collected through laboratory experiments on a dynamic common pool resource game, where, in an infinitely repeated number of rounds (i.e., game ended randomly), individuals made decisions about whether to exert a high or a low effort level to extract resources. Experiments were conducted using the student sample (consent provided and ethics approved) at the University of Hawai**ʻ**i at Mānoa. A total of 8 sessions, 2 for each of the 4 treatments, were run with exactly 20 participants within a session. Individuals made decisions in groups of 10. Communication between any participant was not allowed. A session is randomly assigned (1) to vary whether the inflow of resources at the beginning of each round is high or low, and (2) to allow participants to either financially punish or socially punish defectors. A financial punishment resulted to a loss in profit for the punished while a social punishment displayed the words “You have extracted too much! You're being greedy!” on the computer screen of the punished. Individuals were assigned subject ID numbers and interacted using their subject IDs. The data is useful in understanding how resource inflow and type of punishment affects individual resource extraction behavior. The data could also be combined with other publicly available common pool resource datasets for a meta-analysis on individual behavior in the commons.

**Specifications Table**

**Table:** 

Subject	Microeconomics Ecology Behavioral Economics

Specific subject area	Dynamic common pool resource experiment on inflow level and punishment type.
Type of data	Table (33 columns and 3060 rows)
How the data were acquired	Data was acquired through economic laboratory experiments run using z-Tree. Participants came from the student sample at the University of Hawai**ʻ**i at Mānoa. The experimental instructions and the comprehension check / review questions can be found as supplementary material in the original research article. Participants were randomly assigned into one of eight sessions. Within a session, a participant was randomly assigned a subject ID number. Participants interacted via the computer, could not communicate with other participants, and where only known by their subject ID number.
Data format	Raw Created
Description of data collection	Data was collected using the student sample at the economic laboratory at the University of Hawai**ʻ**i at Mānoa. Students were randomly invited to sessions. All invited students were non-economics majors. Sessions were randomly assigned treatments.
Data source location	Institution: University of Hawai**ʻ**i at Mānoa City: Honolulu State: Hawai**ʻ**i Country: USA
Data accessibility	Repository name: Mendeley Data Data identification number:10.17632/c2z95m5gty.1 Direct URL to data: https://data.mendeley.com/datasets/c2z95m5gty[1]
Related research article	A.L. Abatayo, J. Lynham, Resource Booms and Group Punishment in a Coupled Social-Ecological System, Ecological Economics, 206 (2023), 107730. https://doi.org/10.1016/j.ecolecon.2022.107730[2]

## Value of the Data

• The data is useful in understanding how resource inflow and type of punishment affects individual resource extraction behavior.
• The data is useful for academics, researchers, and policymakers interested in how the interaction between resource inflows and the types of punishment affect cooperation in the commons.
• The data can provide insights on the effectiveness of financial and social punishment on individual cooperative behavior.
• The data can be further exploited to understand how resource dynamics and previous individual extraction and punishment behavior affects present extraction decisions.
• The data can be combined with other publicly available common pool resource datasets for a meta-analysis on individual behavior in the commons.

## 1 Objective

The data was collected for two reasons. First, the data is used to empirically test the predictions of the theoretical model created by Lade, et al. [3] that showed how resource inflows affected extractive behavior of resource users. Second, the data is used to further examine whether extractive behavior of resource users is moderated by different forms of group punishment (i.e., financial vs. social punishment), and how punishment is itself influenced by changes in resource inflows. The data, along with this article, allows the reproduction and replication of all statistical analyses performed for the original article [2].

## 2 Data Description

### 2.1 Downloading and Reading the Data

The data is available for download at Mendeley Data [1]. From the Mendeley Data website, click on “Download All 49 KB” to download a zip file containing the following files: a ReadMe and a Codebook in text format, 5 Stata do-files, and a dataset in DTA format. The total size of the zip file is 49 KB (when unzipped, file size is 324 KB), of which 256 KB is the dataset (“alldata.dta”).

We provide the entire dataset in DTA format. The dataset was created and analyzed using Stata/MP 13.0 for Mac (64-bit Intel). It is compatible with any Stata/MP or Stata/SE versions 13.0 and higher for both Mac and Windows. The data, “alldata.dta”, can also easily be imported in R using the library “haven”. More information regarding the structure of the data is provided below.

To run all the do-files in Stata, open “00 RunMe.do” and change the directory of the main folder (i.e., Line 44) to point to where the unzipped folder that was downloaded from Mendeley Data is located. Lines 25 – 27 of the file “00 RunMe.do” contains a list of files that needed to be installed first. To install these files, remove the asterisk before each line. Save the updated file and then run the entire “00 RunMe.do”. This should automatically install all needed programs and run all analyses for the related article.

### 2.2 Data Structure

The dataset is in long-format with 33 variables. Table 1 below provides a list of included variables, their storage type, display format, and a short description. Summary statistics for each of these variables can be found in “Codebook.txt”. Variables appear as columns and observations appear as rows. The dataset has a total of 3060 observations. The variable “subject_id” provides the unique subject identifier while the variable “sesid” provides the unique session identifier. With 8 sessions and exactly 20 subjects per session, the data contains the decisions of 160 individuals.

**Table 1:** List of variables.

Item Nos.	Variable Name	Storage Type	Display Format	Variable Description
1	Session	str11	%11s	Session Date and Time
2	Subject	byte	%10.0g	Subject in Treatment
3	Group	byte	%10.0g	Group in Treatment
4	Match	byte	%10.0g	Match in Treatment
5	Block	byte	%10.0g	Block Within a Match
6	Round	byte	%10.0g	Rounds in a Block
7	Period	byte	%10.0g	Rounds in a Treatment
8	HighestRound	byte	%10.0g	Highest Paid Round in Match So Far
9	R	double	%10.0g	Initial Resource Amount in Round
10	subject_id	float	%9.0g	Subject ID
11	sesid	float	%9.0g	Session ID
12	financial	float	%9.0g	Financial Punishment Dummy
13	social	float	%9.0g	Social Punishment Dummy
14	low	float	%9.0g	Low Inflow Dummy
15	high	float	%9.0g	High Inflow Dummy
16	strategyA	byte	%10.0g	Strategy A Choice Dummy
17	strategyB	byte	%10.0g	Strategy B Choice Dummy
18	strategyA_old	byte	%10.0g	Strategy A Choice, Prev. Round
19	strategyB_old	byte	%10.0g	Strategy B Choice, Prev. Round
20	countA	byte	%10.0g	Nos. Strategy A Choices in Round
21	countB	byte	%10.0g	Nos. Strategy B Choices in Round
22	message	byte	%10.0g	Social Punish Dummy
23	punish	byte	%10.0g	Financial Punish Dummy
24	badmess	float	%9.0g	Social Punish, Prev. Strategy B
25	badpun	float	%9.0g	Financial Punish, Prev. Strategy B
26	countmessage	byte	%10.0g	Nos. Social Punish in Round
27	countpunish	byte	%10.0g	Nos. Financial Punish in Round
28	message_old	byte	%10.0g	Social Punish, Prev. Round
29	punish_old	byte	%10.0g	Financial Punish, Prev. Round
30	profit	double	%10.0g	Individual Profit in Round
31	age	byte	%10.0g	Age of Participant
32	gender	byte	%10.0g	Gender of Participant
33	studentype	byte	%23.0g	Student Type of Participant

### 2.3 Participant Demographics

The dataset has an almost equal number of females to males (i.e., 58.53% are females and 41.47% are males). Most of the participants are freshmen and sophomore undergraduates in engineering and the natural sciences (i.e., individuals who may have taken “game theory” in the past were excluded during recruitment). Average participant age is 22 – 23 years old, with a minimum age of 18 and a maximum age of 58. All participants were recruited from a database of possible student participants. We discuss the details of this database below, under the subsection “Recruitment”.

### 2.4 Decision Variables

The individual decision variables are labelled “strategyA”, “strategyB”, “message”, and “punish”. The variable “strategyA” is equal to 1 (labelled, “Yes”) if an individual decides to exert a low effort level, and 0 (labelled, “No”) otherwise while the variable “strategyB” is equal to 1 (labelled, “Yes”) if an individual decides to exert a high effort level, and 0 (labeled, “No”) otherwise. The variable “message” is equal to 1 (labelled “Yes”) if an individual decides to socially punish, and 0 (labeled, “No”) otherwise while the variable “punish” is equal to 1 (labelled “Yes”) if an individual decides to financially punish, and 0 (labelled, “No”) otherwise. Table 2 shows the summary statistics of these four variables.

**Table 2:** Summary statistics for decision variables.

Variable	Obs.	Mean	Std. Dev.	Min	Max
strategyA	3060	0.402	0.490	0	1
strategyB	3060	0.598	0.490	0	1
message	3060	0.134	0.341	0	1
punish	3060	0.146	0.354	0	1

All variables that end with “_old” at the end are variables that take the previous round's values. For instance, the variable “strategyA_old” pertains to whether an individual picked the lower effort level in the previous round. Hence, the first round of every block is a missing observation. The first round of every block is identified in the dataset as “Round==1” or “Period==1”, “Period==10”, and “Period==20”. As can be seen in Table 3, means and standard deviations for the variables that end with “_old” remain quite similar to the means and standard deviations of the original decision variables.

**Table 3:** Summary statistics for previous round's decision variables.

Variable	Obs.	Mean	Std. Dev.	Min	Max
strategyA_old	2720	0.404	0.491	0	1
strategy_old	2720	0.595	0.491	0	1
message_old	2720	0.135	0.342	0	1
punish_old	2720	0.150	0.357	0	1

Related to these decision variables are the “count” variables. These are variables in the dataset that start with the word “count” (i.e., countA, countB, countmessage, and countpunish). The “count” variables give the number of individuals within the current group of 10 who decided to implement a particular strategy. For instance, “countA” are number of individuals who picked strategyA in the current round. Like the decision variables, the “count” variables are automatically generated by the software used for the experiment. A summary statistics table for these “count” variables is available in Table 4 below. More information regarding these variables and what type of decisions individuals had to make are discussed in the section on Experimental Design.

**Table 4:** Summary statistics for “count” variables.

Variable	Obs.	Mean	Std. Dev.	Min	Max
countA	3060	4.019	1.709	0	9
countB	3060	5.980	1.709	1	10
countmessage	3060	3.167	1.660	0	9
countpunish	3060	1.941	1.676	0	8

In our experiment, we restrict the number of individuals who can punish to individuals who picked a low effort level. We also restrict punishment to only the individuals who picked a high effort level. If an individual picks a low effort level, “strategyA” is equal to 1 and “strategyB” is equal to 0. If an individual picks a high effort level, “strategyA” is equal to 0 and “strategyB” is equal to 1. The variables, “countA” and “countB”, are the number of individuals, within a match in each round of a session, who picked the low effort and the high effort levels, respectively. As such “countmessage” are the number of “strategyA” individuals in the Social Punishment treatment who decide to punish and “countpunish” are the number of “strategyA” individuals in the Financial Punishment treatment who decide to punish.

### 2.5 Created Variables

Apart from the “_old” variables described above, there are 3 types of created variables in the dataset (i.e., these are variables that the authors created as opposed to variables that were automatically generated by software used to run the experiment). The first type are treatment indicator variables (i.e., variables that either take a value of 0 or 1). There are four created treatment indicator variables in the dataset: “low”, “high”, “financial”, and “social”. The variables are equal to 1, if an observation falls under the low treatment, high treatment, financial treatment, social treatment, or high-financial treatment, respectively; and 0 otherwise. The second type of created variables are also indicator variables but are not treatment related. These are the “bad” variables, composed of “badmess” and “badpun”. The variable “badmess” are participants who were socially punished for picking a high effort level in the previous round while the variable “badpun” are participants who were financially punished for picking a high effort level in the previous round. The last type of created variables are numerical identifiers, composed of “sesid” and “subject_id”. The former is a unique identifier per session and the latter is a unique identifier per individual across all sessions. Hence, 12 out of 33 variables were created.

## 3 Experimental Design, Materials, and Methods

### 3.1 Experimental Design

**Fig. 1:**
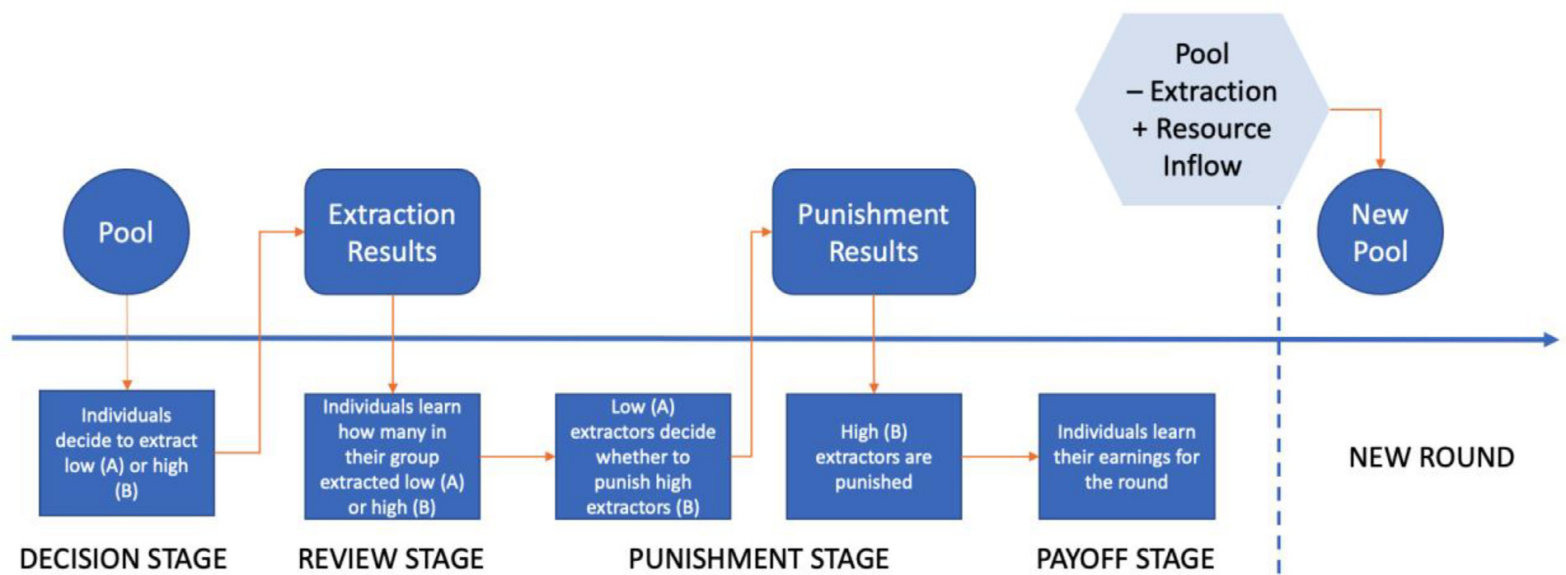
Flow of experiment.

To examine how individual decisions change given differences in resource inflows and types of punishment, we run laboratory experiments at the University of Hawai**ʻ**i at Mānoa. The setup of our experiment follows closely the theoretical work of Lade, et al. [3]. In their setup, individuals can decide to extract using either low or high effort levels, where a high effort level means a higher extraction amount and hence, a higher individual profit. High extraction, however, also implies lower remaining resources. In each round, the amount of resources available for harvest is equal to the amount of resources left in the previous round plus a constant resource inflow minus a function that captures resource decay plus. A mathematical formulation of this can be found in Lade, et al. [3]. The profit function that was shown to the experiment participants can be found in the experiment instructions, which is provided as a supplementary material of the original research article [2]. Fig. 1 below shows the general flow of the experiment. At the end of each round, participants are shown how many individuals in their group chose which effort levels, their profits, and the number of tokens that were added to the token pool for the next period (see Fig. 3c for the actual payoff screen). Participants were told that they will see all these when they were given the experiment instructions.

**Fig. 2:**
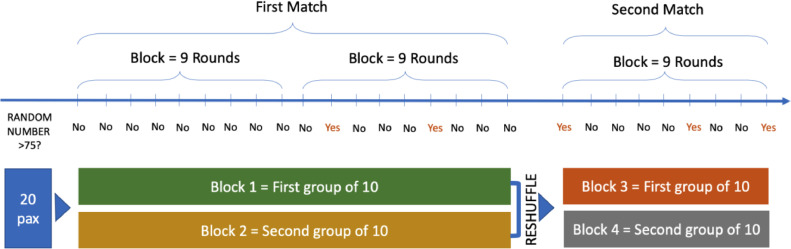
Implementation of an infinitely repeated game and matching protocol.

*Notes:* A match ends when a random number greater than 75 is drawn. Individuals only know when a match ends after block of 9 rounds. Individuals will keep playing blocks of 9 rounds until a random number greater than 75 is drawn.

In Lade, et al. [3], participants made repeated decisions within the same group for an infinite number of rounds. To make this experimentally tractable, we follow the procedures of Frechette and Yuskel [4] for implementing infinitely repeated games in a lab. In our experiment, individuals make repeated decisions within the same group for several rounds. Exactly 20 individuals were recruited to a session. At the start of the experiment, participants were randomly divided into 2 groups of 10 each and made decisions within this group of 10 for a block of 9 rounds. If a random number higher than 75 was generated anytime during this block of 9 rounds, the match ends, and individuals are reshuffled into another group of 10, where they play another block of 9 rounds with the new individuals they are matched with. If no random number higher than 75 was generated anytime during the block of 9 rounds, the group of 10 played for anther block of 9 rounds. This repeats until a number 75 or higher is generated in at least one round. In the experiment, we call a group of 10 individuals a “match”. Within each session, there are exactly 2 matches. Fig. 2 below illustrates how rounds form into blocks and how blocks form into matches. It also illustrates our matching protocol. Individuals are unable to identify which individuals in their new match were part of their old match. The random number generated is the same for all participants within a session. All participants were paid the sum of their profits from both matches for all rounds that did not end. On average, participants earned 15 USD for an hour of doing the experiment.

Lade, et al. [3] provides more detail of the specific functional forms and parameter values that were used in the experiment. We follow closely the setup of Lade, et al. [3], only differing in two main ways: first, we have groups of 10 individuals in a match instead of 50; and second, individuals who choose low effort levels can decide to punish rather than automatically punish like in Lade, et al. [3]. Moreover, while Lade, et al. [3] run simulations on a continuum of resource inflows under a financial punishment regime, our experimental design considers two inflows, high and low, and two forms of punishment, financial and social. Financial punishment led to lower profits for the punished individual while social punishment led to the message “You have extracted too much! You're being greedy!” being displayed on their computer monitors. The number of such message displayed on an individual's screen corresponded to the number of low effort individuals who decided to punish. A summary of our overall experimental design is depicted in Table 5. There were four treatments.

**Table 5:** 2 × 2 Experimental design matrix.

		Resource Inflow	
		Low	High
Punishment	Social	Social, Low	Social, High
	Financial	Financial, Low	Financial, High

### 3.2 Recruitment

Experiment participants were recruited via the Online Recruitment System for Experimental Economics (ORSEE) [5]. To maintain the ORSEE database, the university annually sends an email blast to all students inviting them to be part of the database. Interested students are asked sign-up to this database. Their names, email addresses, year level, and field of study are collected during sign-up. We are unable to match this information to any information collected during the actual experiment.

ORSEE creates sessions with slots for the number of required participants. For this experiment, 8 sessions were created, each with 25 sign-up slots. Because a session in our experiment can only run if it has exactly 20 participants, we over-recruited. If more than 20 individuals showed up, extra participants were given a $5 show-up and asked to sign up for a new session. As is standard in economic experiments, participants were not given any financial incentives during recruitment. They were, however, told that there is a possibility to earn money depending on theirs and other people's decision in the experiment.

From an ORSEE database of ∼5000 students, 100 random non-economics students were sent emails to sign up for the slots in a session. This was done repeatedly, with no replacement, for all 8 sessions.

### 3.3 Implementation

**Fig. 3:**
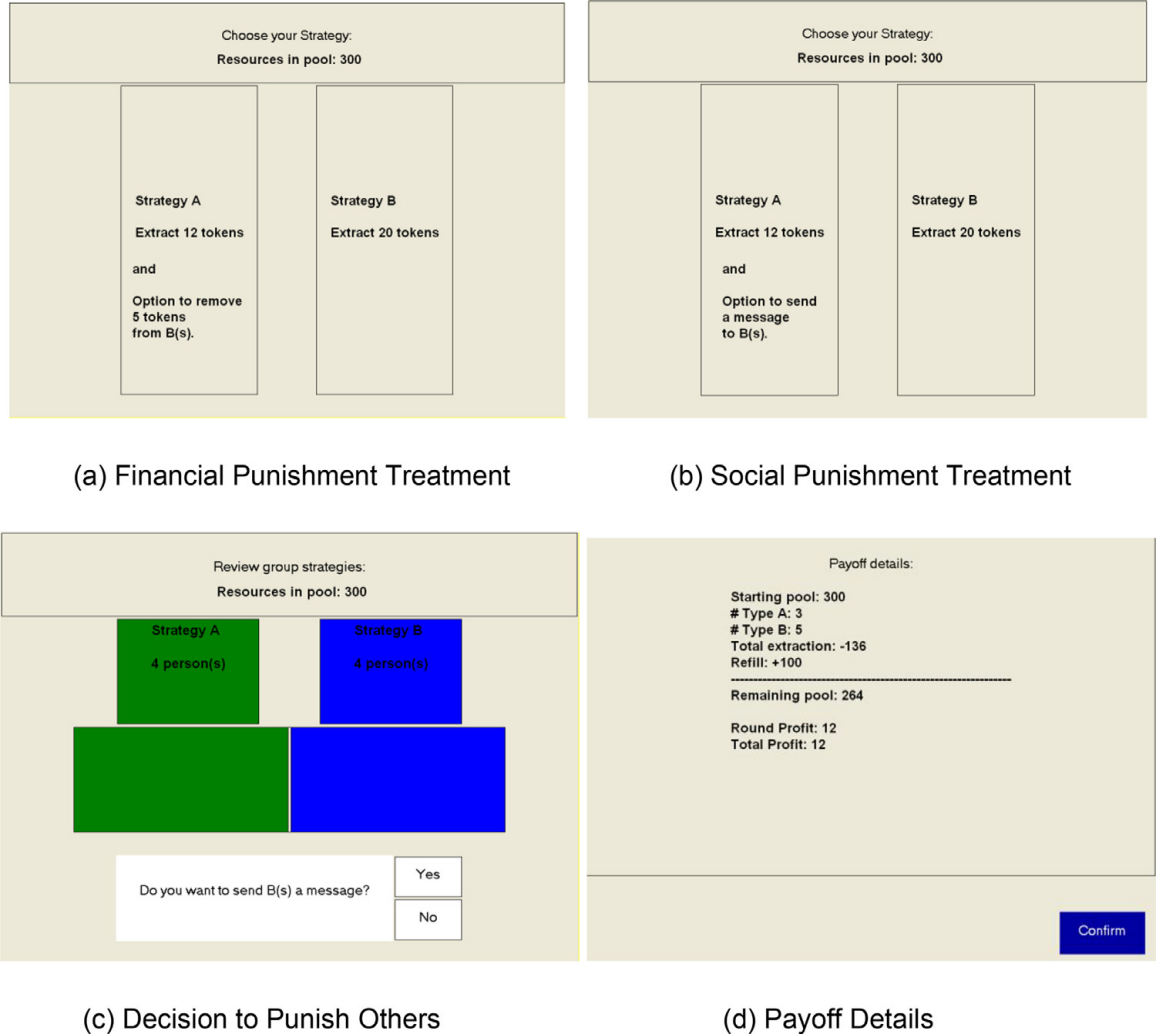
Sample z-tree screen.

*Note:* The screen in Panel (c) is only shown to participants who picked Strategy A under the Social Punishment treatment. Those who picked Strategy B are shown the green and blue boxes, but not the white boxes. Those who picked Strategy A under the Financial Punishment treatment are shown a similar screen, except that they are asked whether they want to remove 5 tokens from those who picked Strategy B rather than whether they want to send a message.

All sessions were conducted using z-Tree [6] and each participant was assigned a computer station with partitions. The partitions made sure that individuals were blocked from their view of all other computer stations. In all sessions, participant consent was taken, experiment instructions were read aloud, and students needed to answer a series of questions about the experiment before being allowed to proceed with the experiment. The latter is not included in the dataset, as these were done via paper-and-pen and not collected by the experimenter. However, to ensure that the participants fully understood the rules of the game, the experiment did not proceed until all participants answered all questions correctly. Participants were not allowed to communicate with one another for the duration of the experiment. Interaction among participants happened via the z-Tree program. A sample of the z-Tree screens that the participants saw during the experiment is show in Fig. 3. At the end of the experiment, participants were called to the back one by one using their subject ID number and then paid their earnings in cash.

### 3.4 Materials

Experiment instructions for each of the four treatment and the comprehension check / review questions can be found in the supplementary material of the original research article [2].

## CRediT authorship contribution statement

**Anna Lou Abatayo:** Conceptualization, Methodology, Software, Investigation, Writing – original draft, Writing – review & editing, Formal analysis, Visualization, Data curation, Project administration. **John Lynham:** Conceptualization, Methodology, Supervision, Writing – review & editing, Formal analysis, Validation, Funding acquisition.

## Declaration of Competing Interest

The authors declare that they have no known competing financial interests or personal relationships that could have appeared to influence the work reported in this paper.

## Data Availability

Dataset for “Resource Booms and Group Punishment in a Coupled Social-Ecological System” (Original data) (Mendeley Data).
